# Microarray-based characterization of differential gene expression during vocal fold wound healing in rats

**DOI:** 10.1242/dmm.018366

**Published:** 2015-01-15

**Authors:** Nathan V. Welham, Changying Ling, John A. Dawson, Christina Kendziorski, Susan L. Thibeault, Masaru Yamashita

**Affiliations:** 1 Department of Surgery, Division of Otolaryngology, University of Wisconsin School of Medicine and Public Health, Madison, WI 53792, USA; 2 Department of Biostatistics and Medical Informatics, University of Wisconsin School of Medicine and Public Health, Madison, WI 53706, USA

**Keywords:** Fibrosis, Larynx, Scar formation, Tissue repair, Transcriptome

## Abstract

The vocal fold (VF) mucosa confers elegant biomechanical function for voice production but is susceptible to scar formation following injury. Current understanding of VF wound healing is hindered by a paucity of data and is therefore often generalized from research conducted in skin and other mucosal systems. Here, using a previously validated rat injury model, expression microarray technology and an empirical Bayes analysis approach, we generated a VF-specific transcriptome dataset to better capture the system-level complexity of wound healing in this specialized tissue. We measured differential gene expression at 3, 14 and 60 days post-injury compared to experimentally naïve controls, pursued functional enrichment analyses to refine and add greater biological definition to the previously proposed temporal phases of VF wound healing, and validated the expression and localization of a subset of previously unidentified repair- and regeneration-related genes at the protein level. Our microarray dataset is a resource for the wider research community and has the potential to stimulate new hypotheses and avenues of investigation, improve biological and mechanistic insight, and accelerate the identification of novel therapeutic targets.

## INTRODUCTION

Wound healing is a complex biological process that is characterized by a dynamic series of molecular, cellular and extracellular events (Martin, 1997). These events primarily occur at the wound site but are also orchestrated, in part, at the organ and organism levels (Kisseleva and Brenner, 2008; Mori et al., 2005; Song et al., 2010). The wound healing process is generally conceptualized as consisting of at least three overlapping phases (Gurtner et al., 2008): inflammation (granulocyte and leukocyte infiltration, inflammatory factor secretion), fibroblast and epithelial cell proliferation [provisional extracellular matrix (ECM) deposition, reepithelialization, tissue contraction] and remodeling (ECM reorganization, crosslinking and maturation). In different biological contexts, wound healing can result in a number of outcomes ranging from complete regeneration with restoration of uninjured morphology and function (e.g. fetal wounds) to impaired healing resulting in chronic pathology (e.g. diabetic wounds) (Colwell et al., 2008; Occleston et al., 2010; Schultz et al., 2011; Sullivan et al., 1995). Furthermore, evidence suggests that both the local injury response and eventual repair outcome differ as a function of tissue type. For example, injured adult skin heals more slowly, and with more scar formation, than adult oral mucosa (Schrementi et al., 2008; Szpaderska et al., 2003) and, under permissive conditions, the regeneration-privileged adult liver (Periwal et al., 2014).

The vocal fold (VF) mucosa is a specialized tissue with distinctive repair and regeneration needs. Unlike other upper airway mucosae, it is routinely subject to high-frequency oscillation driven by aerodynamic forces, resulting in compressive, tensile and shear stresses (Gunter, 2004; Titze, 1988). Its ability to generate voice is predominantly a function of favorable tissue viscoelasticity, which in turn is conferred by the organization and structure of its ECM (Gray et al., 2000; Gray et al., 1999). Following injury, the matrix typically undergoes disordered repair, resulting in impaired phonation (Benninger et al., 1996; Choi et al., 2012; Welham et al., 2011a; Welham et al., 2007) and, in humans, significant morbidity (Cohen et al., 2006; Cohen et al., 2012).

Most existing research in VF wound healing is based on a small set of candidate genes, proteins, signaling molecules and cell types, adopted from work in skin and other mucosal systems. System-level datasets are emerging in other areas of VF biology (Thibeault et al., 2003; Welham et al., 2013; Welham et al., 2011b) and show potential in generating new hypotheses and model refinements, as well as identifying new disease biomarkers and therapeutic candidates. Here, using a previously validated rat injury model (Ling et al., 2010a; Tateya et al., 2005; Tateya et al., 2006b), expression microarray technology and an empirical Bayes analysis approach (Kendziorski et al., 2003), we generated a VF-specific transcriptome dataset to better capture the system-level complexity of VF wound healing and serve as a resource for the wider research community. We characterized transcript-level differential expression in injured and experimentally naïve VF mucosae over time, pursued functional enrichment analyses to refine and add greater biological definition to the previously proposed temporal phases of VF wound healing, and validated the expression and localization of a subset of previously unidentified repair- and regeneration-related genes at the protein level.

## RESULTS

### Within-time-point analysis

**Fig. 1.**
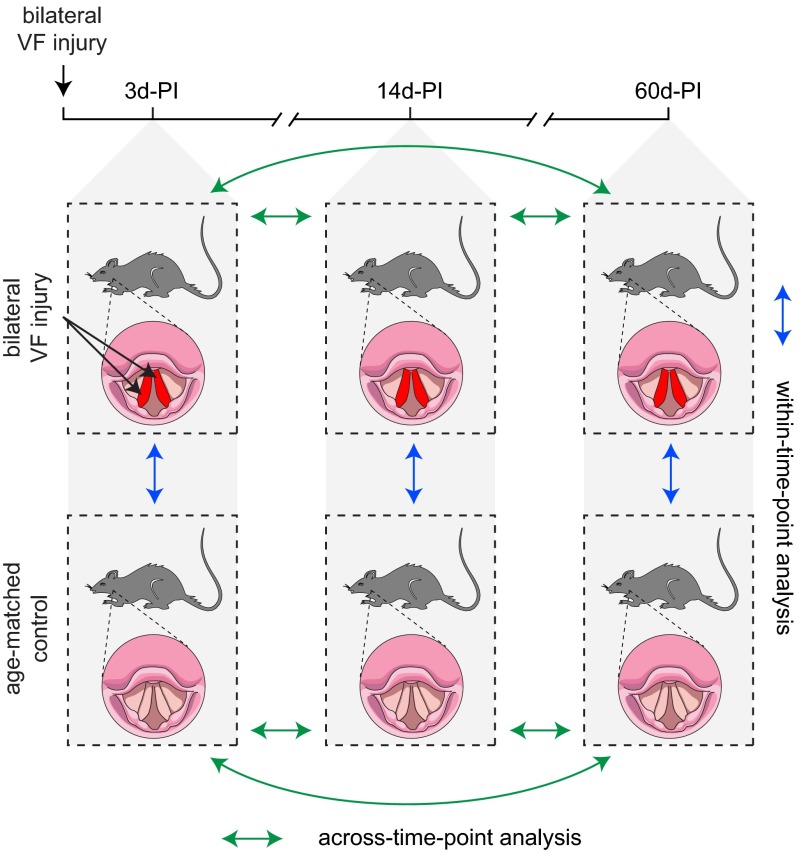
**Experimental design and analysis strategy.** A set of 4-month-old rats received bilateral vocal fold (VF) mucosal stripping injuries, followed by tissue harvest at 3, 14 and 60 days post-injury (d-PI). A parallel set of experimentally naïve age-matched rats were used as controls. Blue arrows indicate the primary comparisons of interest in the within-time-point analysis; green arrows indicate the primary comparisons of interest in the across-time-point analysis.

**Fig. 2.**
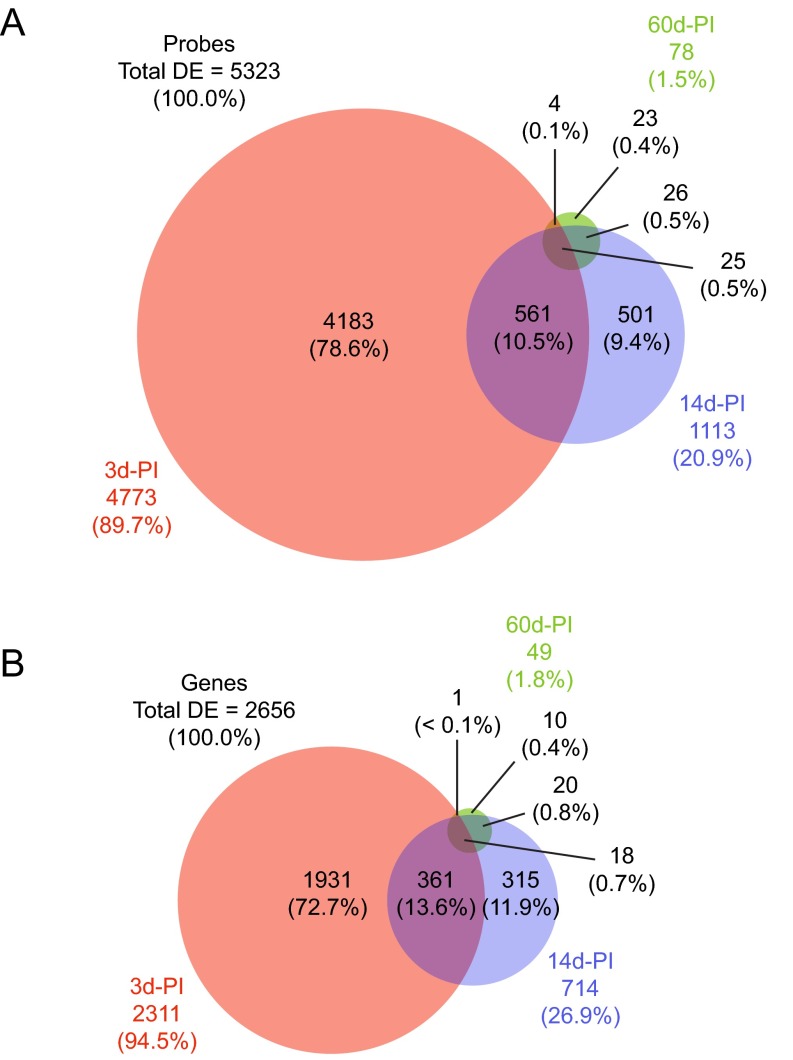
**Within-time-point analysis summary.** Venn diagrams illustrating the overlap in differentially expressed (DE) probes (A) and genes (B) at 3, 14 and 60 days post-injury (d-PI) compared to age-matched controls. Thresholding was performed using a >0.95 posterior probability of differential expression. Owing to rounding errors, not all percentages add up to 100.0%.

We generated a microarray dataset using rat VF mucosae harvested at three post-injury (PI) time points, and VF mucosae from experimentally naïve age-matched controls (Fig. 1). Injured VF mucosa samples were collected and processed at 3, 14 and 60 days PI, based on previously reported rat data suggesting that these time points represent inflammatory, proliferative and remodeling wound healing phases, respectively (Lim et al., 2006; Ling et al., 2010a; Tateya et al., 2006a; Tateya et al., 2005; Tateya et al., 2006b). Using a ‘within-time-point’ analysis strategy, we first evaluated probes and genes that were differentially expressed across the PI and control conditions at individual time points. We observed an overall decrease in differentially expressed probes and genes with wound healing progression: the numbers of differentially expressed probes and genes were greatest at 3 days PI (4773 probes; 2311 genes) and progressively decreased at 14 days PI (1113 probes; 714 genes) and 60 days PI (78 probes; 49 genes) (Fig. 2). The majority of differentially expressed probes and genes at 3 days PI were exclusive to the 3-day-PI time point (4183 probes; 1931 genes); less than half of the differentially expressed probes and genes at 14 days PI were exclusive to the 14-day-PI time point (501 probes; 315 genes); the minority of differentially expressed probes and genes at 60 days PI were exclusive to the 60-day-PI time point (23 probes; 10 genes). A total of 18 genes, represented by 25 probes, were differentially expressed at all time points.

**RESOURCE IMPACT**

**Background**

Injury to the vocal fold (VF) mucosa initiates a series of wound healing events that can lead to scar formation. Scarred VF mucosa is characterized by disordered tissue biomechanics and vibratory function, which can result in loss of voice (dysphonia) and reduced quality of life. There are currently no effective therapies for the scarred VF mucosa. A significant hindrance to progress in this area is the lack of VF-specific ‘omic’ data, particularly at the level of transcriptomics. The availability of ‘omic’ datasets would help to advance our understanding of the system-level complexity of wound healing as it occurs in the dynamic biomechanical environment of the VF mucosa.

**Results**

In this study, the authors used a previously validated *in vivo* rat model of VF injury. They combined expression microarray technology with an empirical Bayes analysis approach to examine differential gene expression at 3, 14 and 60 days post-injury (PI) compared to experimentally naïve controls. Overall, transcriptional activity was highest at 3 days PI and then tapered over time. Early transcription events at 3 days PI were primarily associated with cell division and proliferation, as well as cell adhesion to the provisional extracellular matrix (ECM). Acute inflammatory activity, first detected at this time point, continued further and was accompanied by the acceleration of ECM-related transcription at 14 days PI. The authors observed clear resolution of inflammation- and proliferation-driven transcriptional activity by 60 days PI, by which time they could detect only ten differentially expressed genes. A gene set that is predominantly associated with muscle differentiation, contractile function and repair exhibited sustained differential expression throughout the entire experimental time course.

**Implications and future directions**

The microarray dataset produced in this study is publically available at the Gene Expression Omnibus (GEO) repository and thus represents a valuable resource for the wider research community with the potential to stimulate new hypotheses and avenues of investigation, improve biological and mechanistic insight, and accelerate the identification of novel therapeutic targets for VF injury and scarring. The analysis offers a more accurate biological definition of the temporal phases of VF wound healing compared to that previously proposed. Future work with this ‘omic’ dataset might include the refinement and validation of existing *in vitro* models, the examination of tissue-specific differences in wound healing outcome and, ultimately, the identification of a therapeutically-relevant transcriptomic signature for scarless tissue regeneration.

### Across-time-point analysis

**Fig. 3.**
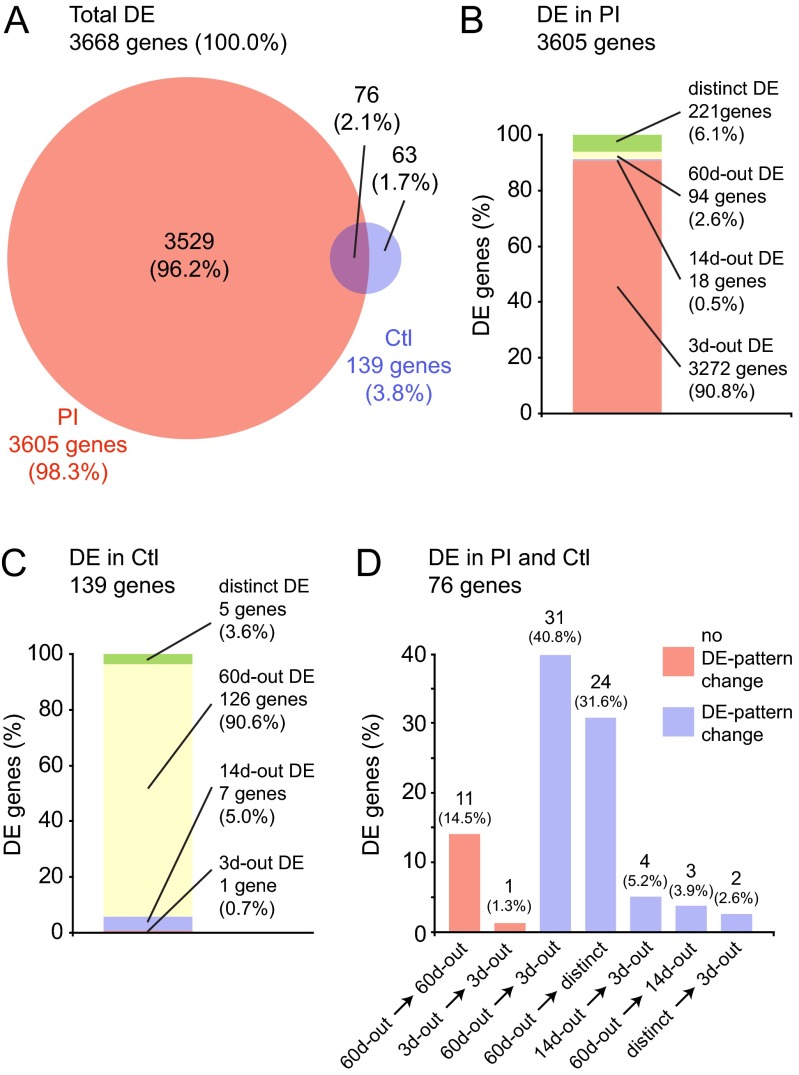
**Across-time-point analysis summary.** (A) Venn diagram illustrating overlap in differentially expressed (DE) genes in post-injury (PI) and control (Ctl) conditions, irrespective of time point. (B) Distribution of differential expression patterns for 3605 differentially expressed genes in the PI condition. The majority of genes (90.8%) exhibited a 3d-out pattern. (C) Distribution of differential expression patterns for 139 differentially expressed genes in the control condition. The majority of genes (90.6%) exhibited a 60d-out pattern. (D) Change in differential expression pattern for 76 differentially expressed genes in both control and PI conditions. The majority of genes (40.8%) changed from a 60d-out pattern in the control condition to a 3d-out pattern in the PI condition. Differential expression patterns are defined in the main text. Thresholding was performed using a >0.95 posterior probability of differential expression. Owing to rounding errors, not all percentages add up to 100.0%.

We also performed a parallel analysis of the gene-level data using an ‘across-time-point’ analysis strategy, focusing on genes that were differentially expressed over time within the PI and control conditions, respectively. Genes identified as differentially expressed within either condition were further categorized into one of four patterns: 3d-outgroup (3d-out; differentially expressed at the 3-day time point compared with at 14 days and 60 days); 14d-out (differentially expressed at the 14-day time point compared with at 3 days and 60 days); 60d-out (differentially expressed at the 60-day time point compared with at 3 days and 14 days); and distinct (differentially expressed across all three time points). A total of 3605 differentially expressed genes were identified in the PI condition (Fig. 3A): the majority (3272 genes; 90.8%) exhibited a 3d-out pattern (Fig. 3B), consistent with predominant transcriptional activity during the inflammatory wound healing phase. In contrast, 139 differentially expressed genes were identified in the control condition (Fig. 3A): the majority (126 genes; 90.6%) exhibited a 60d-out pattern (Fig. 3C), consistent with slow-changing transcriptional activity that might be attributable to maturation or aging. Further analysis of the 76 genes that were differentially expressed in both PI and control conditions revealed that 12 genes (15.8%) showed no change in differential expression pattern across conditions, whereas the majority (31 genes; 41.8%) switched from a 60d-out pattern (the predominant pattern in the control condition) to a 3d-out pattern (the predominant pattern in the PI condition) following injury (Fig. 3D).

### A subset of genes exhibit sustained differential expression throughout the wound healing process

**Fig. 4.**
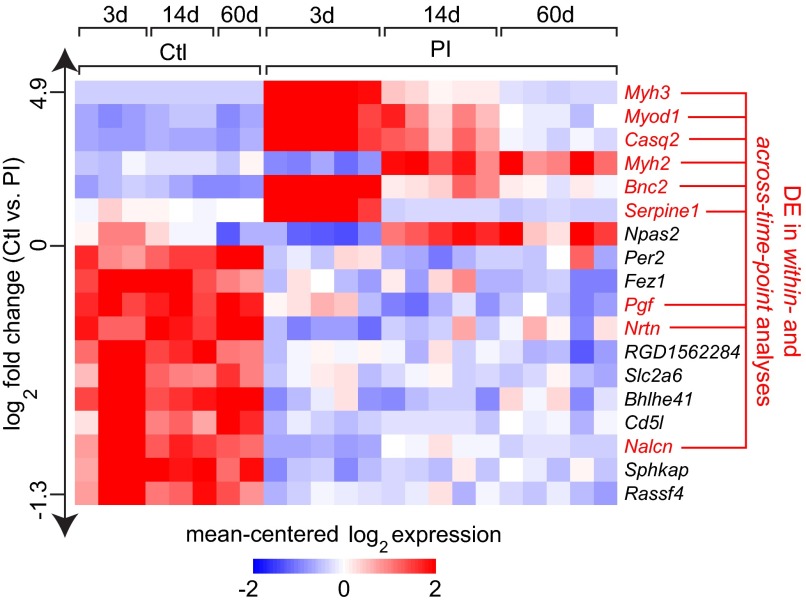
**Heat map summarizing expression data for 18 genes exhibiting differential expression across control and PI conditions at all time points in the within-time-point analysis.** Gene names in red were also differentially expressed (DE) over time in the PI condition [but not the control (Ctl) condition] in the across-time-point analysis. Genes are ranked by log_2_ fold change (mean expression value in PI arrays versus mean expression value in control arrays, irrespective of time point) along the vertical axis. Color intensity represents the mean-centered log_2_ expression within each row. Thresholding was performed using a >0.95 posterior probability of differential expression. Expression values for genes represented by multiple probes reflect the probe with the maximum median for the mean across-array intensity.

Next, we examined the subset of 18 genes that were consistently differentially expressed at 3, 14 and 60 days PI in the within-time-point analysis, compared with genes that were differentially expressed over time in the PI condition but not the control condition in the across-time-point analysis. A total of 9 genes were differentially expressed under both scenarios (Fig. 4), including the muscle-related genes *Myh2*, *Myh3*, *Myod1* and *Casq2*. In addition to exhibiting consistent differential expression across conditions and time points, several genes within this subset showed temporal transcription variation. For example, *Myh3*, *Myod1* and *Casq2*, as well as the fibrinolysis inhibitor *Serpine1* and the basal epithelial cell transcription factor *Bnc2*, showed the highest expression levels at 3 days PI followed by tapering as wound healing progressed, whereas *Myh2* was highly expressed beginning at 14 days PI. Other genes in this subset, such as the angiogenesis promotor *Pgf*, the neurotrophic factor *Nrtn* and the ion channel *Nalcn*, showed more uniform differential expression over the experimental time course.

### Differential expression at 3 days PI

**Fig. 5.**
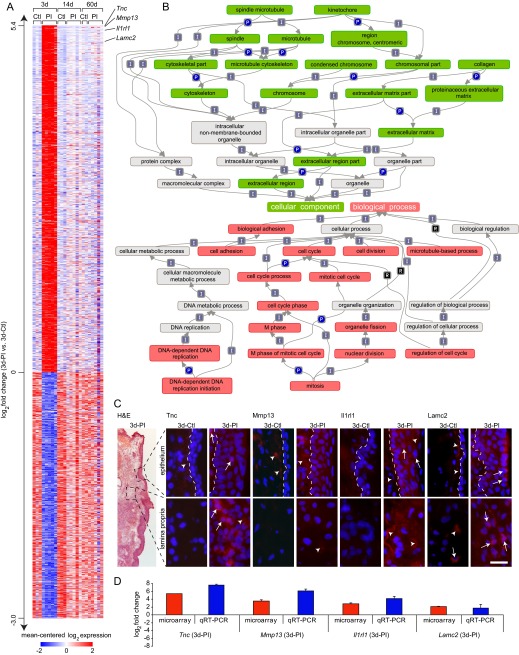
**Expression, enrichment and validation analyses of genes exhibiting differential expression at 3 days PI compared to age-matched controls.** (A) Heat map summarizing expression data for 1931 differentially expressed genes exclusive to the 3-day PI (3d-PI) time point. Genes are ranked by log_2_ fold change [mean expression value in 3d-PI arrays versus mean expression value in age-matched control (3d-Ctl) arrays] along the vertical axis. Color intensity represents mean-centered log_2_ expression within each row. Thresholding was performed using a >0.95 posterior probability of differential expression. Expression values for genes represented by multiple probes reflect the probe with the maximum median for the mean across-array intensity. (B) Hierarchical map summarizing relationships between overrepresented gene ontology terms at 3d-PI compared to 3d-Ctl. Overrepresented biological process terms are red; overrepresented cellular component terms are green; interconnecting terms are gray. Arrows between related terms signify ‘is a’ (I), ‘part of’ (P) or ‘regulates’ (R). Overrepresented terms were enriched for at least 10 differentially expressed genes and surpassed a *z*-score threshold of 5. No overrepresented molecular function terms were identified at this time point. (C) Images showing the morphology of the VF mucosa and immunovalidation of proteins corresponding to four representative gene transcripts of interest at 3d-PI. Frozen VF mucosa coronal sections were stained with H&E, anti-tenascin C (Tnc), anti-matrix metalloproteinase 13 (Mmp13), anti-interleukin 1 receptor-like 1 (Il1rl1) or anti-laminin γ2 (Lamc2) antibodies (red), and the nuclear dye DAPI (blue). Black dashed boxes in the H&E-stained image indicate the approximate anatomic orientation of all immunostained images. White dashed lines in the upper set of immunostained images indicate the boundary between the epithelium (right) and superficial lamina propria (left). Arrows indicate positively labeled cells; arrowheads indicate positive extracellular signals. Scale bar: 300 μm (H&E-stained image); 30 μm (immunostained images). (D) qRT-PCR-based validation of the four representative gene transcripts highlighted in A and analyzed at the protein level in C (mean±s.e.m.).

Of the 1931 genes exclusively differentially expressed at 3 days PI compared to control, 1130 were upregulated in the PI condition and 801 were downregulated in the PI condition (Fig. 5A). Enrichment analysis using the Gene Ontology (GO) annotations (Gene Ontology Consortium, 2013) revealed a cluster of overrepresented biological process terms associated with cell adhesion, cell cycle and mitotic processes, as well as cellular component terms associated with the cytoskeleton, chromosomes and ECM (Fig. 5B). We identified no overrepresented molecular function terms at 3 days PI.

We selected four highly upregulated genes for immunovalidation and localization at the protein level using immunohistochemistry (IHC) (Fig. 5C), alongside validation at the transcript level using quantitative real-time PCR (qRT-PCR) (Fig. 5D). The extracellular glycoprotein transcript *Tnc* was upregulated 41.6-fold in the PI condition compared to control; IHC revealed a corresponding increase in cellular and extracellular tenascin C immunosignals in the lamina propria. The matrix metalloproteinase (specifically, collagenase) transcript *Mmp13* was upregulated 11.9-fold in the PI condition compared to control; IHC revealed a corresponding increase in extracellular matrix metalloproteinase 13 immunosignals in the subepithelium and superficial lamina propria. The Toll-interleukin receptor transcript *Il1rl1* (also known as *St2*) was upregulated 6.7-fold in the PI condition compared to control; IHC revealed a corresponding increase in cellular interleukin 1 receptor-like 1 immunosignals in the epithelium (consistent with the membrane-bound receptor isoform of the protein) as well as extracellular immunosignals in the lamina propria (consistent with the soluble isoform of the protein). The epithelium-basement membrane anchoring filament transcript *Lamc2* was upregulated 4.0-fold in the PI condition compared to control; IHC revealed a corresponding increase in cellular laminin γ2 immunosignals in the luminal epithelium and lamina propria.

### Differential expression at 14 days PI

**Fig. 6.**
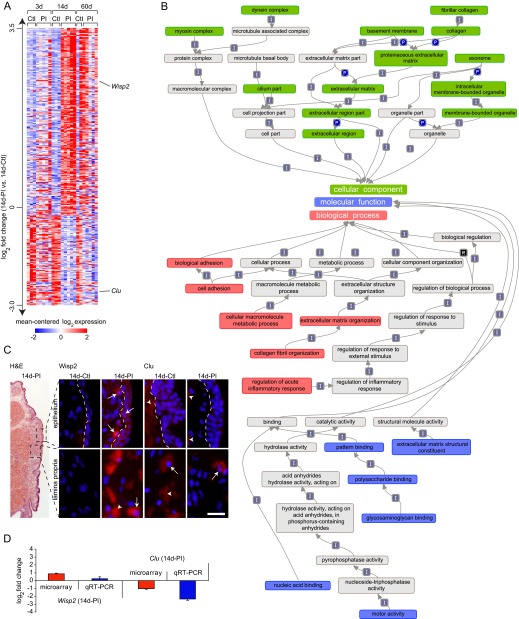
**Expression, enrichment and validation analyses of genes exhibiting differential expression at 14 days PI compared to age-matched controls.** (A) Heat map summarizing expression data for 315 differentially expressed genes exclusive to the 14-day PI (14d-PI) time point. Genes are ranked by log_2_ fold change [mean expression value in 14d-PI arrays versus mean expression value in age-matched control (14d-Ctl) arrays] along the vertical axis. Color intensity represents mean-centered log_2_ expression within each row. Thresholding was performed using a >0.95 posterior probability of differential expression. Expression values for genes represented by multiple probes reflect the probe with the maximum median for the mean across-array intensity. (B) Hierarchical map summarizing relationships between overrepresented gene ontology terms at 14d-PI compared to 14d-Ctl. Overrepresented biological process terms are red; overrepresented cellular component terms are green; overrepresented molecular function terms are blue; interconnecting terms are gray. Arrows between related terms signify ‘is a’ (I), ‘part of’ (P) or ‘regulates’ (R). Overrepresented terms were enriched for at least 10 differentially expressed genes and surpassed a *z*-score threshold of 5. (C) Images showing morphology of the VF mucosa and immunovalidation of proteins corresponding to two representative gene transcripts of interest at 14d-PI. Frozen VF mucosa coronal sections were stained with H&E, anti-Wnt1-inducible-signaling pathway protein 2 (Wisp2) or anti-clusterin (Clu) antibodies (red), and the nuclear dye DAPI (blue). Black dashed boxes in the H&E-stained image indicate the approximate anatomic orientation of all immunostained images. White dashed lines in the upper set of immunostained images indicate the boundary between the epithelium (right) and superficial lamina propria (left). Arrows indicate positively labeled cells; arrowheads indicate positive extracellular signals. Scale bar: 300 μm (H&E-stained image); 30 μm (immunostained images). (D) qRT-PCR-based validation of the two representative gene transcripts highlighted in A and analyzed at the protein level in C (mean±s.e.m.).

Of the 315 genes exclusively differentially expressed at 14 days PI compared to control, 213 were upregulated in the PI condition and 102 were downregulated in the PI condition (Fig. 6A). Enrichment analysis revealed overrepresented biological process terms associated with collagen fibril organization and the regulation of the acute inflammatory response, as well as continued overrepresentation of the cell adhesion term, first identified at 3 days PI (Fig. 6B). Overrepresented molecular function and cellular component terms were also consistent with substantial ECM-related transcriptional activity (e.g. fibrillar collagen, basement membrane, glycosaminoglycan binding), as well as modulation of the axoneme and myosin and dynein complexes.

We selected one upregulated and one downregulated gene for validation using IHC (Fig. 6C) and qRT-PCR (Fig. 6D). The matricellular signaling transcript *Wisp2* was upregulated 1.8-fold in the PI condition compared to control; IHC revealed a corresponding increase in cellular Wnt1-inducible-signaling pathway protein 2 immunosignals in the epithelium and lamina propria. The phagocyte recruiter, complement and apoptosis inhibitor *Clu* was downregulated 2.0-fold in the PI condition compared to control; IHC revealed a corresponding decrease in cellular and extracellular clusterin immunosignals in the lamina propria

### Differential expression at 60 days PI

**Fig. 7.**
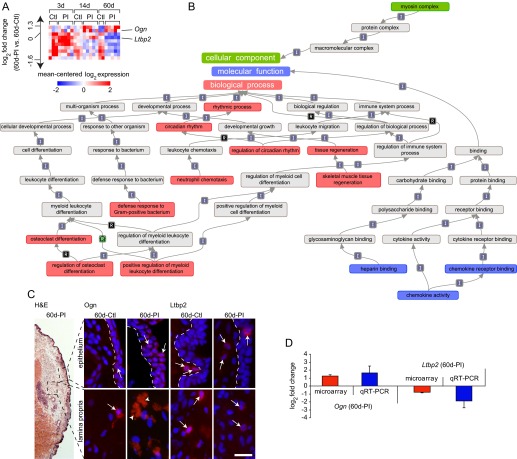
**Expression, enrichment and validation analyses of genes exhibiting differential expression at 60 days post-injury compared to age-matched controls.** (A) Heat map summarizing expression data for 10 differentially expressed genes exclusive to the 60-day PI (60d-PI) time point. Genes are ranked by log_2_ fold change [mean expression value in 60d-PI arrays versus mean expression value in age-matched control (60d-Ctl) arrays] along the vertical axis. Color intensity represents mean-centered log_2_ expression within each row. Thresholding was performed using a >0.95 posterior probability of differential expression. Expression values for genes represented by multiple probes reflect the probe with the maximum median for the mean across-array intensity. (B) Hierarchical map summarizing relationships between overrepresented gene ontology terms at 60d-PI compared to 60d-Ctl. Overrepresented biological process terms are red; overrepresented cellular component terms are green; overrepresented molecular function terms are blue; interconnecting terms are gray. Arrows between related terms signify ‘is a’ (I), ‘part of’ (P) or ‘regulates’ (R). Overrepresented terms were enriched for at least 10 differentially expressed genes and surpassed a *z*-score threshold of 5. (C) Images showing morphology of the VF mucosa and immunovalidation of proteins corresponding to two representative gene transcripts of interest at 60d-PI. Frozen VF mucosa coronal sections were stained with H&E, anti-osteoglycin (Ogn) or anti-latent transforming growth factor-β binding protein 2 (Ltbp2) antibodies (red), and the nuclear dye DAPI (blue). Black dashed boxes in the H&E-stained image indicate the approximate anatomic orientation of all immunostained images. White dashed lines in the upper set of immunostained images indicate the boundary between the epithelium (right) and superficial lamina propria (left). Arrows indicate positively labeled cells; arrowheads indicate positive extracellular signals. Scale bar: 300 μm (H&E-stained image); 30 μm (immunostained images). (D) qRT-PCR-based validation of the two representative gene transcripts highlighted in A and analyzed at the protein level in C (mean±s.e.m.).

Of the 10 genes exclusively differentially expressed at 60 days PI compared to control, two were upregulated in the PI condition and eight were downregulated in the PI condition (Fig. 7A). Enrichment analysis revealed overrepresented biological process terms associated with skeletal muscle regeneration, cell differentiation, chemotaxis and the bacteria-induced defense response, molecular function terms associated with chemokine activity and heparin binding, and one cellular component term associated with the myosin complex, as first identified at 14 days PI.

We selected one upregulated and one downregulated gene for validation using IHC (Fig. 7C) and qRT-PCR (Fig. 7D). The extracellular proteoglycan transcript *Ogn* was upregulated 2.3-fold in the PI condition compared to control; IHC revealed a corresponding increase in cellular osteoglycin immunosignals in the epithelium and extracellular immunosignals in the lamina propria. The growth factor binding protein and extracellular microfibril assembly transcript *Ltbp2* was downregulated 1.7-fold in the PI condition compared to control; IHC revealed a corresponding decrease in cellular latent transforming growth factor-β-binding protein 2 immunosignals within the lamina propria, as well as occasional cellular signals in the epithelium.

## DISCUSSION

Despite key differences in tissue environment, VF wound healing has traditionally been viewed as being comparable to that of skin and other non-VF mucosae. This assumption might not hold, however, as although many classic wound healing elements are conserved across organ systems, certain parameters, such as isoform-specific signaling within the transforming growth factor-β cytokine superfamily (Chang et al., 2014; Schrementi et al., 2008), appear to direct tissue-specific differences in healing outcome. Given these observations and the specialized phenotype of VF mucosa, we used a previously validated rat injury model (Ling et al., 2010a; Tateya et al., 2005; Tateya et al., 2006b) and expression microarray technology to better define VF wound healing at the transcriptome level. We observed clear patterns in VF-specific transcriptional activity as wound healing progressed, identified corresponding changes in presumed biological function through GO-based enrichment analyses, and validated a subset of repair- and regeneration-related genes at the protein level. Our microarray dataset is a resource for the wider research community with the potential to stimulate new hypotheses and avenues of investigation, improve biological and mechanistic insight, and accelerate the identification of novel therapeutic targets.

By profiling the transcriptome, our dataset provides improved insight into key biological processes that occur during VF wound healing. The analysis of overall (and relative) transcriptional activity at each time point gives an indication of the level of regional biological activity occurring as VF wound healing progresses. The greatest transcriptional activity (measured by both the total number of differentially expressed genes and their mean fold change) was evident at the earliest (3 days PI) time point, in both the within- and across-time-point analyses. Furthermore, in the within-time-point analysis, the majority of genes that were differentially expressed over time in the control group (presumably due to maturation or aging) exhibited a switch from the 60d-out to the 3d-out differential expression pattern following VF injury, suggesting that their role in wound healing overrides their homeostatic function(s). These observations of dominant transcriptional activity at 3 days PI are consistent with previous descriptions of the acute VF injury response involving rapid cellular mobilization and infiltration (Branski et al., 2005; Ling et al., 2010a; Ling et al., 2010b), delivery of cytokines and other signaling molecules (Lim et al., 2006; Ohno et al., 2009; Welham et al., 2008), establishment of a provisional ECM (Branski et al., 2005; Tateya et al., 2006b) and initiation of reepithelialization (Chang et al., 2014; Ling et al., 2011). The sharp reduction in differential expression seen at 60 days PI (i.e. 10 differentially expressed genes, exhibiting log_2_ fold changes of −1.6 to 1.3) is consistent with previous work showing that the majority of wound healing events are completed by this time point in the rat VF (Tateya et al., 2005). Reports in other injury systems (e.g. skin) have suggested that ECM remodeling can continue beyond 1 year after injury (Gurtner et al., 2008); some of these remodeling events, such as fibrous protein crosslinking, prolination and glycosylation, occur at the protein and post-translational levels and are therefore best examined with complementary assays.

As noted above, the conceptualization of VF wound healing into inflammatory, proliferative and remodeling phases (the timing and duration of which were used to select our experimental time points) is based on the extrapolation of classic descriptions in other model systems, as well as limited transcript, protein and histological data from injured VF tissue (Lim et al., 2006; Ling et al., 2010a; Tateya et al., 2006a; Tateya et al., 2005; Tateya et al., 2006b). It is apparent from our rat VF microarray data, however, that these phases are not discrete. Early transcription events (i.e. at 3 days PI) were primarily associated with cell division and proliferation, as well as cell adhesion to the provisional ECM; acute inflammatory activity continued at 14 days PI and was accompanied by the acceleration of ECM-related transcription. This overlapping of biological events across wound healing phases is consistent with current understanding in the general literature (Gurtner et al., 2008; Martin, 1997) and reinforces the dynamic complexity of tissue repair in the VF microenvironment. We observed clear resolution of this inflammation- and proliferation-driven transcriptional activity by 60 days PI, by which time there were just 10 differentially expressed genes. Sampling at additional post-injury time points would provide improved temporal characterization of the VF injury response, particularly during the abovementioned transitional periods that connect overlapping wound healing phases.

A subset of transcripts exhibited sustained differential expression throughout the entire experimental time course. Notably, several members of this gene set, such as *Myh2*, *Myh3*, *Myod1* and *Casq2*, are associated with muscle differentiation, contractile function and repair. This finding suggests that thyroarytenoid muscle repair is a relatively slow process compared to that of the VF mucosa, an observation that is further supported by the enriched skeletal muscle regeneration ontology term seen at 60 days PI. The detection of muscle-specific gene transcription in our dataset is not surprising given that: (1) our surgical injury procedure involved progressively stripping the VF mucosa to achieve muscle exposure, and (2) prior proteomic studies have consistently reported contaminating muscle proteins in microdissected VF mucosa (Welham et al., 2011b; Welham et al., 2013).

In future work, it would be valuable to compare these transcriptomic data with those generated from VF mucosal wounds of varying severity (Imaizumi et al., 2014; Mau et al., 2014), including in humans (Hirano et al., 2009), as many clinical patients undergo mucosal resections that are more conservative than the stripping procedure used in this study. Future studies might also consider the correspondence between select aspects of VF wound healing *in vivo* and the behavior of cultured VF fibroblasts and epithelial cells harvested from naïve, injured or scarred tissue. Classic expression microarray-based work with naïve human dermal fibroblasts, for example, has shown that the *in vitro* response of these cells to serum stimulation is consistent with certain aspects of the initial (i.e. hemostatic) injury response *in vivo* (Iyer et al., 1999); more recent research using this experimental approach suggests that there are distinct gene sets expressed by fibroblasts isolated from the regeneration-privileged oral mucosa, naïve skin and non-healing chronically wounded skin (Peake et al., 2014). The identification of such transcriptomic signatures for biologically relevant conditions in VF mucosa might lead to improved evaluation of emerging and future therapies: specifically, their capacity to drive the injured VF mucosa towards regeneration, rather than fibrosis.

## MATERIALS AND METHODS

### Mucosal injury procedure and tissue harvest

All animal experiments were performed in accordance with the Public Health Service Policy on Humane Care and Use of Laboratory Animals and the Animal Welfare Act (7 U.S.C. et seq.); the animal use protocol was approved by the Institutional Animal Care and Use Committee of the University of Wisconsin-Madison.

Four-month-old Fischer 344 male rats (Charles River, Wilmington, MA) were used for all experiments. Bilateral VF mucosal stripping injuries were created under endoscopic guidance as previously reported (Ling et al., 2010a; Tateya et al., 2005); experimentally naïve age-matched rats were used as controls. Animals were killed and tissue harvested at three time points to capture global expression profiles characteristic of the inflammatory phase (3 days PI), proliferative phase (14 days PI), and maturation and remodeling phase (60 days PI) of wound healing. A total of 20 PI rats (five arrays, *n*=4 pooled animals per array) and 12 control rats (three arrays, *n*=4 pooled animals per array) were reserved for microarray analysis and qRT-PCR validation at each time point (total *n*=96). An additional three PI rats and one control rat were reserved for histological and IHC analyses at each time point (total *n*=12). Our pooling strategy and sample size were based on typical RNA yields and VF mucosal injury-induced transcription changes reported in previous studies (Chang et al., 2014; Chang et al., 2010).

VF mucosa samples intended for RNA isolation were microdissected in an RNase-free environment, immersed in 10 μl RNAlater (Qiagen, Valencia, CA) at 4°C overnight, and then transferred to −80°C. Whole-mount larynges intended for histology and IHC were embedded in optimal cutting temperature (OCT) compound (Tissue Tek, Sakura, Tokyo, Japan), frozen with acetone and dry ice, and stored at −80°C until sectioning. Frozen sections (8 μm thick) were prepared in the coronal plane using a Leica CM-3050S cryostat (Leica, Wetzlar, Germany).

### RNA isolation

Total RNA was isolated using the RNeasy Micro kit (Qiagen) according to the manufacturer’s instructions. RNA yield and integrity were evaluated using a NanoDrop ND-1000 spectrophotometer (NanoDrop, Wilmington, DE), and samples meeting the following three criteria were retained: a concentration above 40 ng/ml, an OD_260:280_ of 1.8–2.0 and an OD_260:230_ above 1.8. Samples were further evaluated using the Agilent 2100 Bioanalyzer and RNA 6000 Pico kit (Agilent, Santa Clara, CA) according to the manufacturer’s instructions. Samples with electropherograms exhibiting sharp 18S and 28S rRNA peaks and no evidence of degradation were retained.

### Microarrays

Total RNA yield in the 60-day PI group was sufficient to run just four of the five arrays intended for this condition. All other arrays and conditions were run as planned. Biotinylated antisense cRNA was prepared by single round *in vitro* amplification of 1.2 μg input RNA using the MessageAmp II-Biotin Enhanced aRNA kit (Ambion, Austin, TX) according to the manufacturer’s instructions (the *in vitro* transcription reaction was performed at 37°C for 14 hours). Polyadenylated RNA controls (Affymetrix, Santa Clara, CA) were spiked into each reaction. Fragmented cRNA sample quality was confirmed by using 2% agarose gel electrophoresis, an Agilent 2100 Bioanalyzer analysis (Pico kit) and hybridization to Affymetrix GeneChip Test3 arrays. Samples were hybridized to Affymetrix GeneChip Rat Genome 230 2.0 arrays at 45°C for 16 hours. Post-processing was performing using the GeneChip Fluidics Station 450, arrays were scanned using the GC3000 G7 scanner and fluorescence intensity data were background-corrected and extracted using Expression Console software (Affymetrix). All hybridization, post-processing and scanning procedures were performed according to Affymetrix protocols; all control parameters for Test3 and rat genome arrays were within manufacturer guidelines. Microarray data have been deposited with the Gene Expression Omnibus (http://www.ncbi.nlm.nih.gov/geo/) under accession number GSE62204.

### qRT-PCR

Reverse transcription was performed using the QuantiTect RT kit (Qiagen) with 300 ng input total RNA per 20 μl reaction, according to the manufacturer’s instructions. Negative controls were prepared without RNA template and without reverse transcriptase. qRT-PCRs were run using the following rat-specific commercial primers (QuantiTect, Qiagen): QT01081297 (*Clu*), QT00178955 (*Il1rl1*), QT00379260 (*Lamc2*), QT00192220 (*Ltbp2*), QT00385686 (*Mmp13*), QT00435015 (*Ogn*), QT00195958 (*Sdha*), QT02340814 (*Tnc*), QT00189840 (*Wisp2*). Reactions were performed on a 7500 Fast Real-Time PCR system (Applied Biosystems, Foster, CA) using the QuantiTect SYBR Green PCR kit (Qiagen). Each 25 μl total volume reaction contained 12.5 μl 2× QuantiTect Master Mix, 2.5 μl 10× QuantiTect Primer Assay and 10 μl cDNA template (diluted 1:10 with nuclease-free H_2_O). Amplifications were performed in MicroAmp Fast Optical 96-well reaction plates with optical adhesive film covers (Applied Biosystems) according to cycling conditions suggested for the Applied Biosystems 7500 instrument in the QuantiTect SYBR Green handbook (initial activation at 95°C for 15 minutes; 40 cycles of 94°C for 15 seconds, 55°C for 30 seconds, 72°C for 30 seconds).

PCR runs were performed in duplicate using cDNA synthesized from the same batch and starting amount of total RNA. Negative controls containing no cDNA template were included for each gene within each PCR run. To avoid the influence of variation in amplification conditions across runs, all reactions for a given gene of interest were performed in the same 96-well plate. Amplification specificity for each gene was confirmed by a single distinct melting curve.

qRT-PCR data were analyzed using the 2^−ΔΔCT^ method (Livak and Schmittgen, 2001). Mean cycle threshold (CT) values from duplicate runs were used as input data (duplicate CT values consistently varied by <0.1). *Sdha*, previously validated as stably expressed in our rat VF injury model (Chang et al., 2010), was used as the reference gene. Data were presented as log_2_-transformed mean±s.e.m. fold change

### Histology and IHC

Routine hematoxylin and eosin (H&E) staining was performed to evaluate overall tissue morphology. Sections intended for IHC were fixed in 4% paraformaldehyde, washed with phosphate-buffered saline (PBS), and incubated with Block-Ace (AbD Serotech, Raleigh, NC) and 5% donkey serum (Jackson ImmunoResearch, West Grove, PA) for 30 minutes to block non-specific binding. Next, sections were sequentially incubated with a primary antibody for 90 minutes followed by a relevant secondary antibody for 60 minutes, with thorough washing between each incubation step. Finally, slides were covered with anti-fade mounting medium containing DAPI (Vectashield, Vector Labs, Burlingame, CA) and coverslips were added. Control sections stained with an isotype control or without the primary or secondary antibody showed no immunoreactivity.

The primary antibodies used were: rabbit anti-osteoglycin (Ogn), clone M-70 (1:50; sc-67170, Santa Cruz Biotechnology, Santa Cruz, CA); goat anti-latent transforming growth factor-β-binding protein 2 (Ltgp2), clone E-18 (1:100; sc-18343, Santa Cruz Biotechnology); rabbit anti-Wnt1-inducible-signaling pathway protein 2 (Wisp2), clone H-74 (1:50; sc-25442, Santa Cruz Biotechnology); rabbit anti-clusterin (Clu), clone H-330 (1:150; sc-8354, Santa Cruz Biotechnology); goat anti-tenascin C (Tnc), clone F-17 (1:50, sc-9872; Santa Cruz Biotechnology); goat anti-laminin γ2 (Lamc2), clone G-16 (1:50; sc-31092, Santa Cruz Biotechnology); goat anti-interleukin 1 receptor-like 1 (Il1rl1), clone C-20 (1:50; sc-18687, Santa Cruz Biotechnology); and goat anti-matrix metalloproteinase 13 (Mmp13), (1:50; AB8120, Millipore, Billerica, MA). The secondary antibodies used were: Texas-Red-conjugated donkey anti-goat- and donkey anti-rabbit-IgG (1:200; Jackson ImmunoResearch).

Brightfield and fluorescent microscopy images were captured using a microscope (E-600, Nikon, Melville, NY) connected to a digital microscopy camera (Olympus DP-71, Center Valley, PA). All images were captured with consistent exposure settings. Representative images from each experimental group were selected for presentation.

### Statistical analysis

Microarray data were analyzed within the R statistical computing environment (R Development Core Team, 2007). Affymetrix probe-level data were preprocessed using Robust Multi-Array Analysis (RMA) (Irizarry et al., 2003), based on evidence of improved precision over default Affymetrix algorithms (Wu and Irizarry, 2004). Probes without a corresponding gene symbol were purged from all gene-level analyses. In cases where multiple probes corresponded to a single gene symbol, we selected the probe with the median mean across-array intensity; in the case of an even number of matched probes, we selected the larger of the two median probe intensities. The resulting normalized data were clustered to check for consistency prior to formal analysis.

Expression analysis was performed using an empirical Bayes approach as implemented in the R package EBarrays (Kendziorski et al., 2003). A lognormal-normal moderated variance (LNNMV) model was fitted to the data; parameter estimates were obtained through 20 iterations of an expectation-maximization (EM) algorithm where convergence was achieved after 10 iterations. We pursued two analysis strategies in parallel: a within-time-point analysis was used to compare PI and control data at individual time points, and an across-time-point analysis was used to evaluate change in expression over time in the PI and control groups, respectively. For all comparisons, thresholding was performed using a >0.95 posterior probability of differential expression, providing false discovery rate control at the 5% level. Heat maps summarizing expression data for differentially expressed gene lists of interest were generated using Matrix2png (Pavlidis and Noble, 2003).

Tests of enrichment through overrepresentation were conducted using the R package allez (Newton et al., 2007), the GO dataset, and genes identified as exclusively differentially expressed at 3, 14 and 60 days PI in the within-time-point analysis. Overrepresented ontology terms required at least 10 distinct differentially expressed genes and a *z*-score >5. Ontology maps were generated using OBO-Edit (Day-Richter et al., 2007).

Initial diagnostic testing was performed using quantile-quantile (QQ) plots of log intensity data versus a standard normal distribution. We further used QQ plots and comparisons of theoretical and empirical densities to evaluate the assumption of a scaled inverse chi-square prior on the gene-specific variances used in the LNNMV model. The diagnostics did not show evidence of an unacceptable model fit.
